# Long noncoding RNA FGF14-AS2 inhibits breast cancer metastasis by regulating the miR-370-3p/FGF14 axis

**DOI:** 10.1038/s41420-020-00334-7

**Published:** 2020-10-12

**Authors:** Yucui Jin, Ming Zhang, Rui Duan, Jiashu Yang, Ying Yang, Jue Wang, Chaojun Jiang, Bing Yao, Lingyun Li, Hongyan Yuan, Xiaoming Zha, Changyan Ma

**Affiliations:** 1 Jiangsu Key Laboratory of Xenotransplantation, Nanjing Medical University, Longmian Road 101, Nanjing, People’s Republic of China; 2 Department of Medical Genetics, Nanjing Medical University, Longmian Road 101, Nanjing, People’s Republic of China; 3 Division of Breast Surgery, The First Affiliated Hospital of Nanjing Medical University, Nanjing, People’s Republic of China; 4 Department of Oncology and Lombardi Comprehensive Cancer Center, Lombardi Comprehensive Cancer Center, Washington, DC USA

**Keywords:** Breast cancer, Breast cancer

## Abstract

Long noncoding RNAs (lncRNAs) have emerged as important regulators in cancers, including breast cancer. However, the overall biological roles and clinical significance of most lncRNAs are not fully understood. This study aimed to elucidate the potential role of a novel lncRNA FGF14-AS2 and the mechanisms underlying metastasis in breast cancer. The lncRNA FGF14-AS2 was significantly downregulated in breast cancer tissues; patients with lower FGF14-AS2 expression had advanced clinical stage. In vitro and in vivo assays of FGF14-AS2 alterations revealed a complex integrated phenotype affecting breast cancer cell migration, invasion, and tumor metastasis. Mechanistically, FGF14-AS2 functioned as a competing endogenous RNA of miR-370-3p, thereby leading to the activation of its coding counterpart, FGF14. Clinically, we observed increased miR-370-3p expression in breast cancer tissues, whereas FGF14 expression was decreased in breast cancer tissues compared to the adjacent normal breast tissues. FGF14-AS2 expression was significantly negatively correlated with miR-370-3p expression, and correlated positively to FGF14 expression. Collectively, our findings support a model in which the FGF14-AS2/miR-370-3p/FGF14 axis is a critical regulator in breast cancer metastasis, suggesting a new therapeutic direction in breast cancer.

## Introduction

Breast cancer is one of the most common malignancies and is a major cause of death among women worldwide^1,2^. Despite significant advances in early diagnosis, surgical intervention, and local and systemic adjuvant therapies, the overall 5-year survival rate remains low. The main causes of poor prognosis in patients with breast cancer are distant metastasis and tumor recurrence^3,4^. Although great efforts have been made to clarify the pathophysiologic mechanisms contributing to the progression of breast cancer, much of it remain unknown. Therefore, it is vital to identify new regulators involved in breast cancer to facilitate the development of novel cancer biomarkers and effective therapeutic strategies.

Long noncoding RNAs (lncRNAs), a subgroup of ncRNAs, comprise >200 nucleotides with little protein-coding potential^5,6^. A growing number of studies have shown that lncRNAs participate in several important biological processes, such as stem cell differentiation, cell senescence, cancer cell migration, metastasis, and apoptosis^7–9^. Although over 95,000 human lncRNAs have been annotated^10^, only a few, such as HOX transcript antisense RNA (HOTAIR) and metastasis-associated lung adenocarcinoma transcript 1 (MALAT1), have been well characterized in various carcinomas^11,12^. LncRNAs can interact with signaling proteins to regulate signal transduction at the post-translational level^13^. They can also serve as molecular decoys and scaffolds or enhancers of transcription, and it is intriguing that a large group of lncRNAs function as competing endogenous RNAs (ceRNAs) that regulate gene expression by sponging microRNAs (miRNAs)^14–16^.

Recently, lncRNAs were reported to play important roles in drug resistance, occurrence, and metastasis of breast cancer. For example, the lncRNA TINCR (terminal differentiation-induced ncRNA) sponges miR-125b, promoting trastuzumab resistance-induced epithelial–mesenchymal transition (EMT) by increasing Snail-1 expression^17^. The lncRNA NKILA [nuclear factor (NF)-κB-interacting LncRNA)] suppresses transforming growth factor-β-induced EMT by blocking NF-κB signaling in breast cancer^18^. The lncRNA PVT1 regulates triple-negative breast cancer through KLF5–β-catenin signaling^19^. Although several lncRNAs with oncogenic or cancer-suppressive functions have been identified in breast cancer, it remains unclear whether other lncRNAs are also involved in breast cancer tumorigenesis and progression. Therefore, it is critically important to investigate other breast cancer-associated lncRNAs and elucidate their biological functions to clarify the pathogenesis of breast cancer.

FGF14-AS2 (Gene ID: 283481), an antisense lncRNA, is transcribed from the opposite strand of the FGF14 (fibroblast growth factor 14) gene on chromosome 13q33. There has been no in-depth study on FGF14-AS2, except that FGF14-AS2 is downregulated in human breast cancer tissues and may be involved in cancer progression and prognosis^20^. However, the specific biological effects and molecular mechanisms of FGF14-AS2 in breast cancer progression remain unclear. Here, we determined that FGF14-AS2 was markedly downregulated in breast cancer tissues, and lower FGF14-AS2 expression was related to advanced clinical stage. Loss- and gain-of-function assays indicated that FGF14-AS2 inhibited breast cancer cell migration and invasion. Moreover, ectopic expression of FGF14-AS2 repressed lung metastasis of breast cancer in vivo. RNA immunoprecipitation (RIP) and luciferase reporter assays indicated that FGF14-AS2 acted as a ceRNA of miR-370-3p, allowing FGF14 upregulation and subsequently suppressing breast cancer metastasis. Collectively, our data suggest that FGF14-AS2/miR-370-3p/FGF14, as a novel regulatory axis, may be a potential therapeutic target in breast cancer.

## Results

### FGF14-AS2 is downregulated in breast cancer tissues and associated with tumor-node-metastasis (TNM) stage

**Fig. 1:**
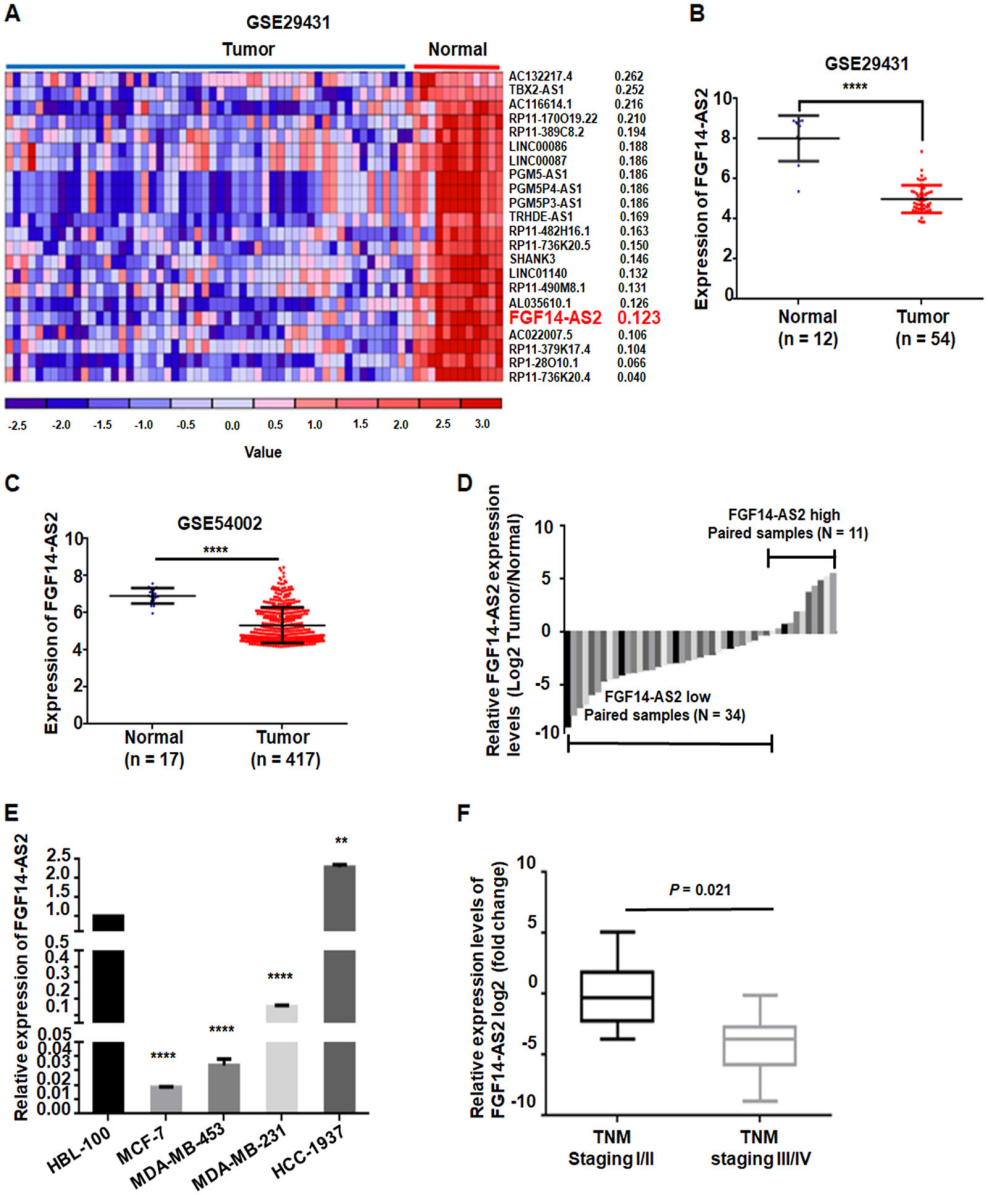
FGF14-AS2 is significantly downregulated in breast cancer and correlated with breast cancer progression. **a** Hierarchical clustering analysis of differentially expressed lncRNAs in breast cancer tissues (Tumor) and non-tumorous samples (Normal) from the GSE29431 dataset (fold change >2; *P* < 0.05). **b** Relative expression of FGF14-AS2 in breast cancer tissues (Tumor) compared with non-tumor samples (Normal) in the GSE29431 dataset. **c** Relative expression of FGF14-AS2 in breast cancer tissues (Tumor) compared with non-tumorous samples (Normal) in the GSE54002 dataset. **d** qRT-PCR determination of relative expression levels of FGF14-AS2 in 45 pairs of breast cancer tissues and corresponding adjacent non-cancerous tissues. **e** qRT-PCR detection of the relative expression levels of FGF14-AS2 in normal breast epithelial cell line (HBL-100) and breast cancer cell lines (MCF-7, MDA-MB-453, MDA-MB-231, and HCC-1937). **f** Relative expression levels of FGF14-AS2 in patients with early-stage (I/II) and advanced-stage (III/IV) breast cancer. Data are the mean ± SD of three independent experiments. **P < 0.01; ****P < 0.0001.

To identify the lncRNAs that may be involved in breast cancer tumorigenesis, we first analyzed breast cancer microarray profile GSE29431 (54 patients with breast cancer, 12 controls) from the Gene Expression Omnibus (GEO) database. Nineteen lncRNAs were upregulated and 75 lncRNAs were downregulated in the breast cancer tissues (fold change >2.0, *P* < 0.05; Fig. 1a and Figure S1). Among the differentially expressed lncRNAs, FGF14-AS2 was significantly lower in breast cancer tissues than in noncancerous tissues (Fig. 1b). Similar results were observed in the GSE54002 dataset (417 patients with breast cancer, 17 controls) (Fig. 1c). To validate the microarray findings, we examined FGF14-AS2 expression levels in 45 paired breast cancer and noncancerous tissues using quantitative real-time reverse transcription PCR (qRT-PCR). FGF14-AS2 was downregulated in most breast cancer tissues (34/45) compared with the paired noncancerous tissues (Fig. 1d). Moreover, FGF14-AS2 expression was lower in three breast cancer cell lines (MCF-7, MDA-MB-453, and MDA-MB-231) compared to the normal breast epithelial cell line HBL-100. However, FGF14-AS2 was highly expressed in HCC-1937 cells (Fig. 1e). This difference may be due to their different source, clinical, and pathological features. We examined the clinicopathological role of FGF14-AS2 and found that FGF14-AS2 expression level correlated negatively with TNM stage (*n* = 45; *P* = 0.021), but was not associated with other factors, including age, estrogen receptor (ER) status, progesterone receptor (PR) status, and HER2 status (Fig. 1f; Table S1). Based on these findings, we speculate that FGF14-AS2 might act as a tumor suppressor gene in breast cancer progression.

### FGF14-AS2 suppresses breast cancer cell migration, invasion, and metastasis

**Fig. 2:**
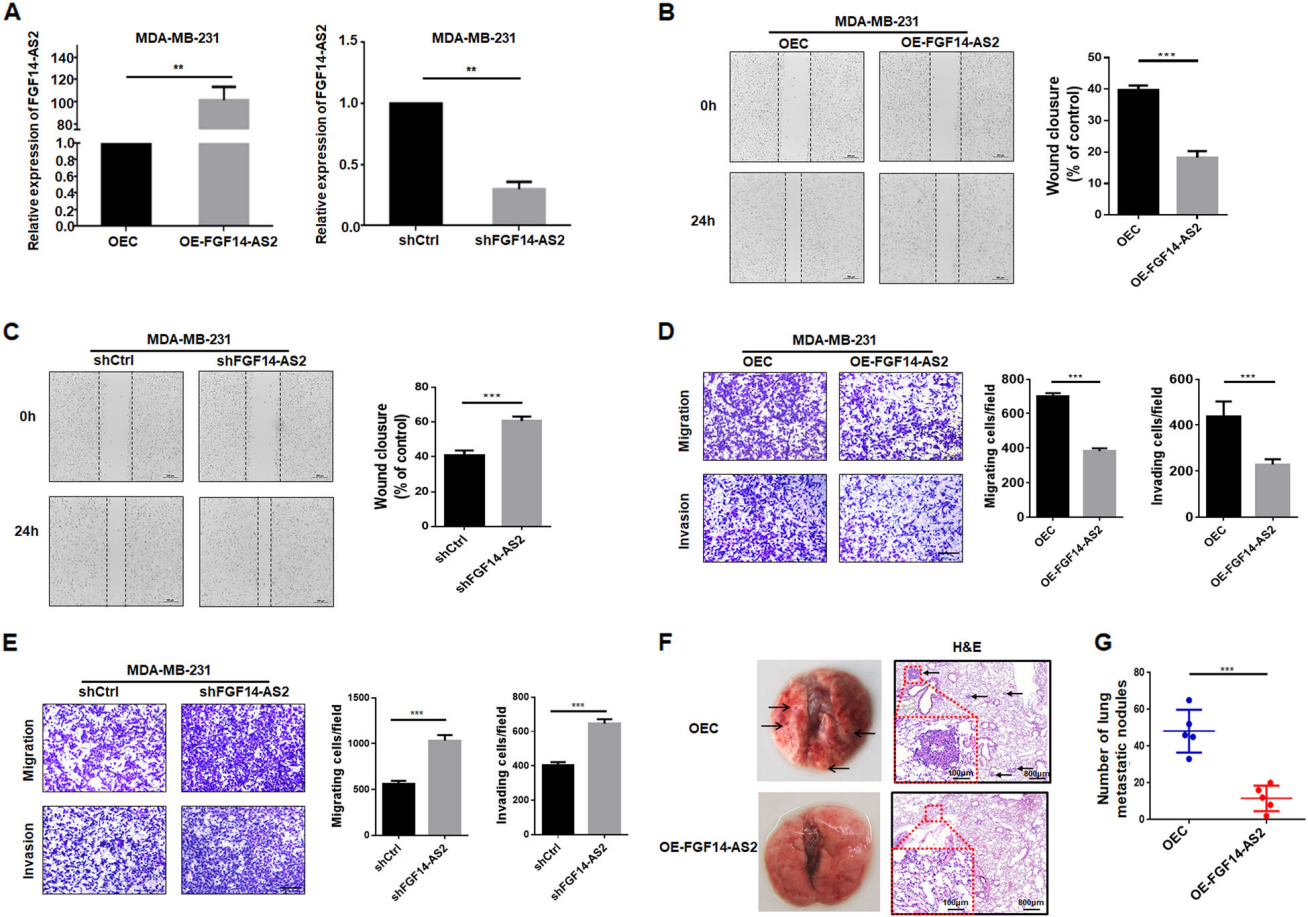
FGF14-AS2 suppresses breast cancer cell migration and invasion in vitro, and tumor metastasis in vivo. **a** FGF14-AS2-overexpressing and knockdown MDA-MB-231 cell lines were established (OEC: overexpression control cells; OE-FGF14-AS2: FGF14-AS2-overexpressing cells; shCtrl: knockdown control cells; shFGF14-AS2: FGF14-AS2 knockdown cells). **b**, **c** Wound healing assay of the migration abilities of FGF14-AS2-overexpressing and knockdown cells. Scale bar: 200 μm. **d**, **e** Transwell assay of the migratory and invasive abilities of FGF14-AS2-overexpressing and knockdown cells. Scale bar: 100 μm. **f** Left: Representative morphology of mouse lung dissected 6 weeks after tail vein injection (*n* = 5 per group). Right: Representative images of H&E-stained dissected mouse lung. Arrows, lung metastatic nodules. **g** Bar graphs showing the numbers of metastatic foci in the lungs per view (*n* = 5 per group). Data are the mean ± SD. **P < 0.01; ***P < 0.001.

To investigate the biological functions of FGF14-AS2 in breast cancer, we performed loss- and gain-of-function studies in MDA-MB-231 cells. Figure 2a shows the ectopic expression and knockdown of FGF14-AS2 in MDA-MB-231 cells. FGF14-AS2 overexpression decreased the migration and invasive abilities of the cells, whereas FGF14-AS2 knockdown presented the opposite effects (Fig. 2b–e). Similar results were obtained in MCF-7 and HCC-1937 cells (Figures S2 and S3). To avoid the possible off-target effect of single short hairpin RNA (shRNA), we repeated the experiments using another two RNA interference targets. Results showed that FGF14-AS2 could be knocked down efficiently, and the migration and invasion of MDA-MB-231 cells were promoted significantly (Figure S4). In addition, CCK-8 assays indicated that FGF14-AS2 knockdown or overexpression had no obvious effects on cell proliferation in breast cancer cells (Figure S5).

To evaluate the effect of FGF14-AS2 on tumor metastasis in vivo, FGF14-AS2-overexpressing MDA-MB-231 cells were transplanted into immunodeficient mice by tail vein injection. Six weeks after injection, the lungs of the FGF14-AS2 group showed a relatively normal appearance and size compared to the control group; the latter exhibited more metastatic nodules, which was confirmed by hematoxylin–eosin (H&E) staining of the lung sections (Fig. 2f). The FGF14-AS2 group had significantly reduced lung metastatic nodules compared with the control group (Fig. 2g). Collectively, these data suggest that FGF14-AS2 suppresses breast cancer cell migration and invasion in vitro and tumor metastasis in vivo.

### FGF14 mediates the tumor-suppressive activity of FGF14-AS2

**Fig. 3:**
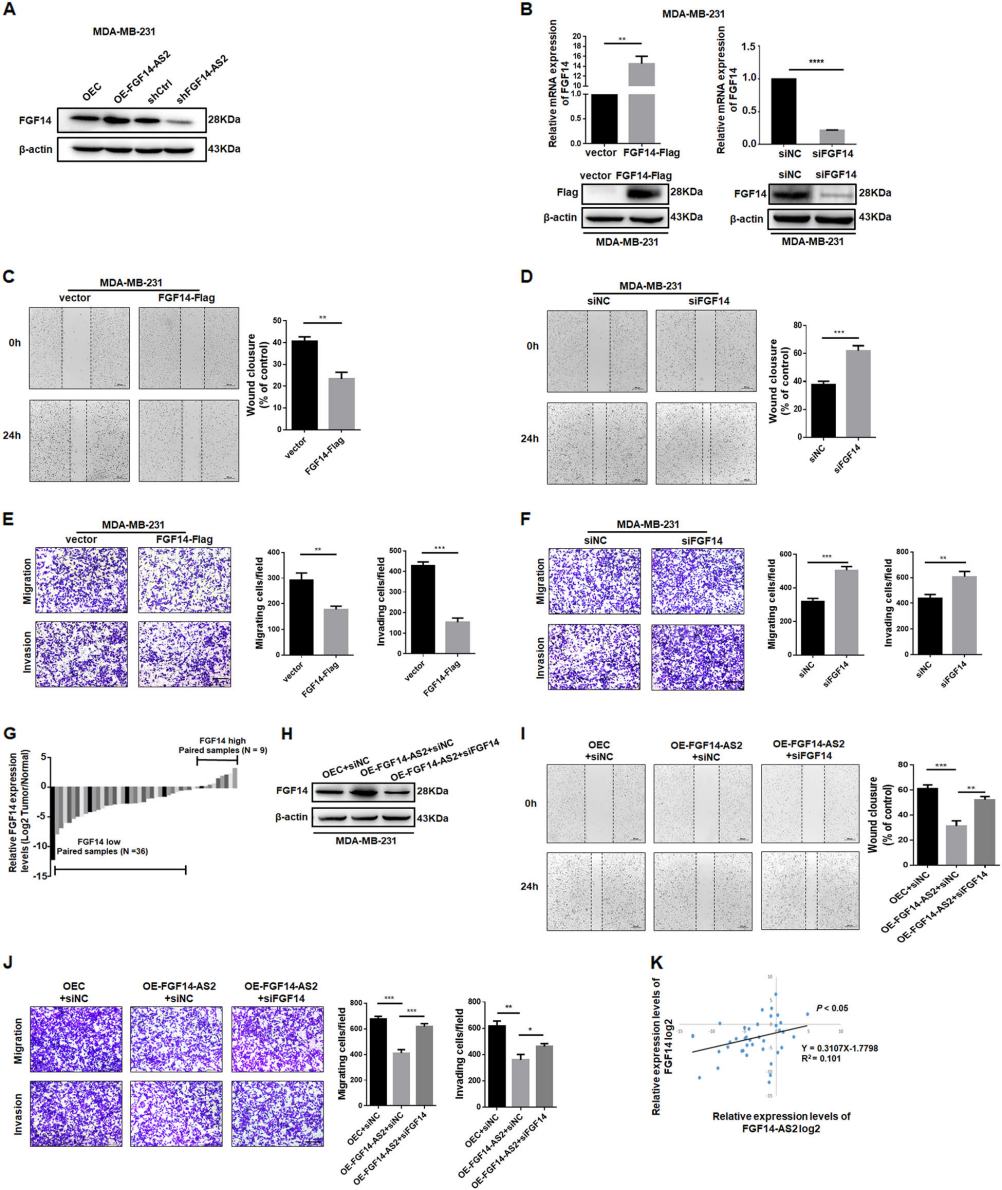
Knockdown of FGF14 partly impairs the FGF14-AS2 inhibitory effect on MDA-MB-231 cell migration and invasion. **a** Western blotting detection of FGF14 expression levels in FGF14-AS2-overexpressing and knockdown cells. **b** qRT-PCR and western blotting detection of FGF14 expression levels in cells transfected with pCMV3-FGF14-Flag or siFGF14. **c**, **d** Wound healing assay of the migration abilities of FGF14-Flag or siFGF14-transfected MDA-MB-231 cells. Scale bar: 200 μm. **e**, **f** Transwell assay of the migratory and invasive abilities of FGF14-Flag or siFGF14-transfected MDA-MB-231 cells. Scale bar: 100 μm. **g** qRT-PCR analysis of FGF14 expression levels in paired breast cancer and corresponding adjacent non-tumor control samples (*n* = 45). **h** Western blotting analysis of FGF14 levels in cells co-transfected with pLVX-FGF14-AS2 and siNC, or with pLVX-FGF14-AS2 and siFGF14. **i** Wound healing assays of the migration abilities of cells co-transfected with pLVX-FGF14-AS2 and siNC, or with pLVX-FGF14-AS2 and siFGF14. Scale bar: 200 μm. **j** Transwell assays of migratory and invasive abilities of cells co-transfected with pLVX-FGF14-AS2 and siNC, or with pLVX-FGF14-AS2 and siFGF14. Scale bar: 100 μm. **k** Spearman correlation analysis of the association between FGF14-AS2 and FGF14 expression levels in 45 breast cancer tissues. Data are the mean ± SD of three independent experiments. *P < 0.05; **P < 0.01; ***P < 0.001; ****P < 0^.^0001.

As the perturbation of antisense RNA can directly or indirectly regulate the expression of sense genes^21,22^, we investigated the effect of FGF14-AS2 on the expression of its coding counterpart, FGF14. Western blotting analysis showed that FGF14-AS2 overexpression increased FGF14 expression, while FGF14-AS2 knockdown reduced it in MDA-MB-231 cells (Fig. 3a). Consistently, qRT-PCR and immunohistochemical staining of mouse lungs showed increased FGF14 mRNA and protein abundance in the FGF14-AS2 group compared with the control group (Figure S6). To investigate the roles of FGF14 in breast cancer, we transfected pCMV3-FGF14-Flag or si-FGF14 into MDA-MB-231 cells to overexpress or knock down FGF14 expression, which was assessed by qRT-PCR and western blotting (Fig. 3b). Wound healing and Transwell assays revealed that FGF14 overexpression inhibited cell migration and invasion significantly, whereas FGF14 knockdown promoted it (Fig. 3c–f). We also analyzed the FGF14 expression levels in paired breast cancer and noncancerous tissues. FGF14 was markedly decreased in most breast cancer tissues (36/45) compared to the adjacent normal tissues (Fig. 3g). These data suggest that FGF14 may function as a tumor suppressor gene in breast cancer.

To determine whether FGF14 mediates the tumor-suppressive effects of FGF14-AS2 in breast cancer cells, we transfected si-FGF14 into FGF14-AS2-overexpressing MDA-MB-231 cells. FGF14-AS2 overexpression led to increased FGF14 expression; however, transient transfection of si-FGF14 repressed FGF14 expression (Fig. 3h). In addition, subsequent functional experiments showed that FGF14 knockdown partly impaired the FGF14-AS2 inhibitory effects on MDA-MB-231 cell migration and invasion (Fig. 3i, j). Moreover, FGF14-AS2 expression levels correlated positively to FGF14 expression in breast cancer tissues (Fig. 3k). Collectively, these data suggest that FGF14-AS2 suppresses breast cancer cell migration and invasion, at least in part, through upregulation of FGF14.

### FGF14-AS2 acts as a sponge of miR-370-3p

**Fig. 4:**
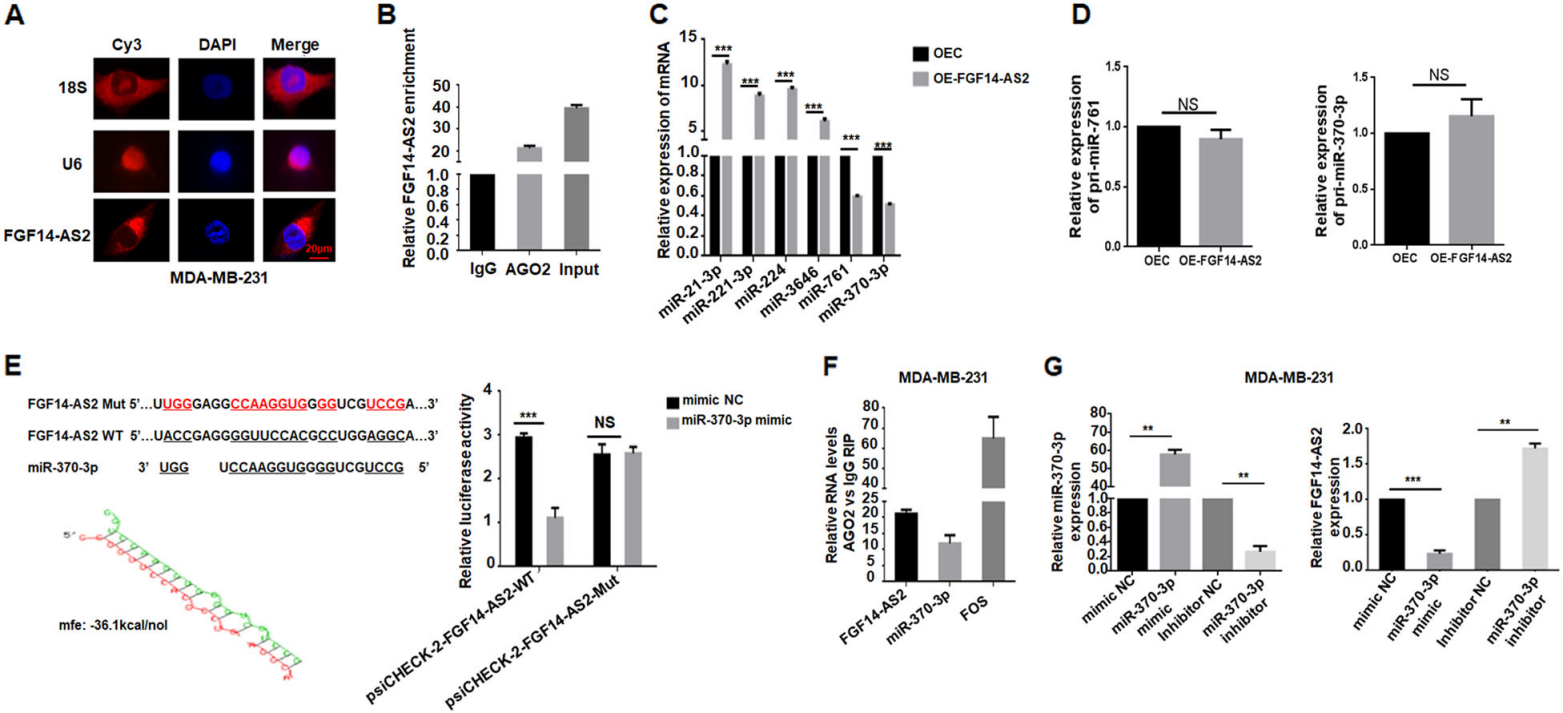
FGF14-AS2 interacts with miR-370-3p. **a** FISH detection of FGF14-AS2 subcellular localization in MDA-MB-231 cells. *18S* and *U6* were modified by cyanine 3 (Cy3, red). *18S* was used as a cytoplasmic marker; *U6* was used as a nuclear marker. The nucleus was stained by DAPI (blue). Scale bar: 20 μm. **b** RIP experiments were performed in MDA-MB-231 cells and the coprecipitated RNAs were subjected to qRT-PCR for FGF14-AS2. The fold enrichment of FGF14-AS2 in AGO2 pellet is relative to its matching IgG control. **c** qRT-PCR was performed to detect the miRNA expression in FGF14-AS2-overexpressing MDA-MB-231 cells. **d** qRT-PCR was performed to detect the expression levels of pri-miR-761 and pri-miR-370-3p in FGF14-AS2-overexpressing MDA-MB-231 cells. **e** Left: The predicted miR-370-3p binding sites in FGF14-AS2 and the designed mutant sequence (WT: wild type; Mut: mutant). Right: Luciferase reporter assay in HEK 293T cells transfected with psiCHECK-2-FGF14-AS2 (WT or Mut) and miR-370-3p mimic. **f** RNA levels in immunoprecipitates were presented as fold enrichment in AGO2 relative to IgG immunoprecipitates. FOS was used as a positive control. **g** qRT-PCR detection of miR-370-3p and FGF14-AS2 expression levels in MDA-MB-231 cells transfected with miR-370-3p mimic, miR-370 inhibitor, and their respective controls. **P < 0.01; ***P < 0.001. NS, no significant difference.

The coding capability of FGF14-AS2 was determined by the online tool Coding Potential Assessment Tool^23^ (http://lilab.research.bcm.edu/cpat/), which indicated that FGF14-AS2 had no protein-coding ability (Figure S7). We used fluorescence in situ hybridization (FISH) to determine the subcellular localization of FGF14-AS2, and found that FGF14-AS2 was more abundant in the cytoplasm (Fig. 4a), suggesting that FGF14-AS2 might regulate target gene expression at the post-transcriptional level. Indeed, RIP revealed that FGF14-AS2 bound directly to AGO2 (Argonaute-2), a component of the RNA-induced silencing complex involved in miRNA-mediated mRNA repression (Fig. 4b). These findings suggest that FGF14-AS2 may act as a ceRNA by competitively binding miRNAs, regulating the expression of the target gene in the cytoplasm. To verify this hypothesis, we used online bioinformatics databases to identify potential miRNAs that can not only target FGF14, but also have FGF14-AS2 binding sites. A total of six miRNAs (miR-21-3p, miR-221-3p, miR-224, miR-3646, miR-761, and miR-370-3p) were initially predicted to bind to both FGF14-AS2 and the FGF14 3′-untranslated region (3′-UTR). Then, qRT-PCR was performed to confirm whether FGF14-AS2 overexpression could downregulate the expression of these miRNAs. Only miR-761 and miR-370-3p were significantly downregulated in the FGF14-AS2-overexpressing group as compared with the control group (Fig. 4c). In addition, FGF14-AS2 overexpression could not alter the expression levels of pri-miR-761 and pri-miR-370-3p (Fig. 4d). Dual-luciferase reporter assay revealed that miR-370-3p could repress luciferase activity by targeting the FGF14 3′-UTR, while miR-761 did not alter the luciferase activity (Fig. 6a and Figure S8). Accordingly, miR-370-3p was selected for further investigation.

To validate the direct binding between FGF14-AS2 and miR-370-3p, we constructed wild-type (FGF14-AS2-WT) and miR-370-3p binding site mutant-type FGF14-AS2 (FGF14-AS2-Mut) luciferase reporters. As expected, miR-370-3p overexpression reduced the FGF14-AS2-WT luciferase activity significantly, but not that of FGF14-AS2-Mut (Fig. 4e). In addition, RIP assay showed that FGF14-AS2 and miR-370-3p were preferentially enriched in AGO2-containing miRNA ribonucleoproteins relative to the control immunoglobulin G (IgG) (Fig. 4f). We also evaluated the effect of miR-370-3p on FGF14-AS2 expression. MiR-370-3p overexpression downregulated FGF14-AS2 expression, whereas miR-370-3p knockdown caused the opposite changes (Fig. 4g), indicating mutual regulation between miR-370-3p and FGF14-AS2.

**Fig. 5:**
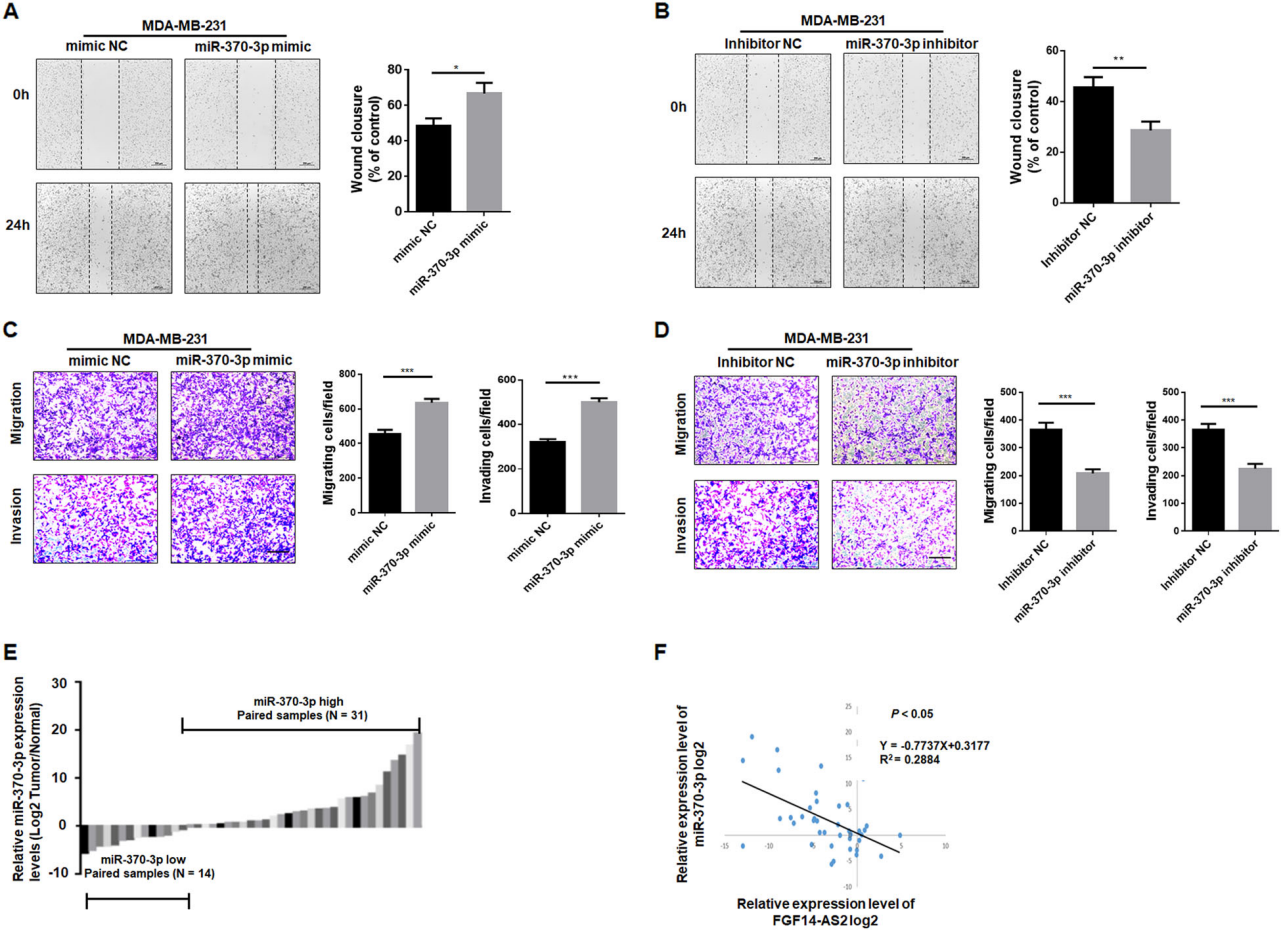
MiR-370-3p promotes breast cancer cell migration and invasion in vitro. **a**, **b** Wound healing assay of the migration abilities of MDA-MB-231 cells transfected with miR-370-3p mimic, miR-370-3p inhibitor, or their respective controls. Scale bar: 200 μm. **c**, **d** Transwell assay of the migratory and invasive abilities of MDA-MB-231 cells transfected with miR-370-3p mimic, miR-370-3p inhibitor, or their respective controls. Scale bar: 100 μm. **e** qRT-PCR analysis of miR-370-3p expression in breast cancer tissues and the corresponding adjacent non-cancerous tissues (*n* = 45). **f** Spearman correlation analysis of the association between FGF14-AS2 and miR-370-3p expression in 45 breast cancer tissues. Data are the mean ± SD of three independent experiments. *P < 0.05; **P < 0.01; ***P < 0.001.

To date, miR-370-3p plays different roles in several types of tumors^24–27^; however, its role in breast cancer requires further exploration. Enforced expression of miR-370-3p promoted cell migration and invasion, whereas knockdown of endogenous miR-370-3p suppressed cell migration and invasion (Fig. 5a–d). In addition, miR-370-3p expression was significantly higher in breast cancer tissues (31/45) compared to adjacent normal tissues (Fig. 5e). Importantly, miR-370-3p expression was negatively associated with FGF14-AS2 expression in breast cancer tissues (Fig. 5f).

### FGF14 is a target of miR-370-3p and indirectly regulated by FGF14-AS2

**Fig. 6:**
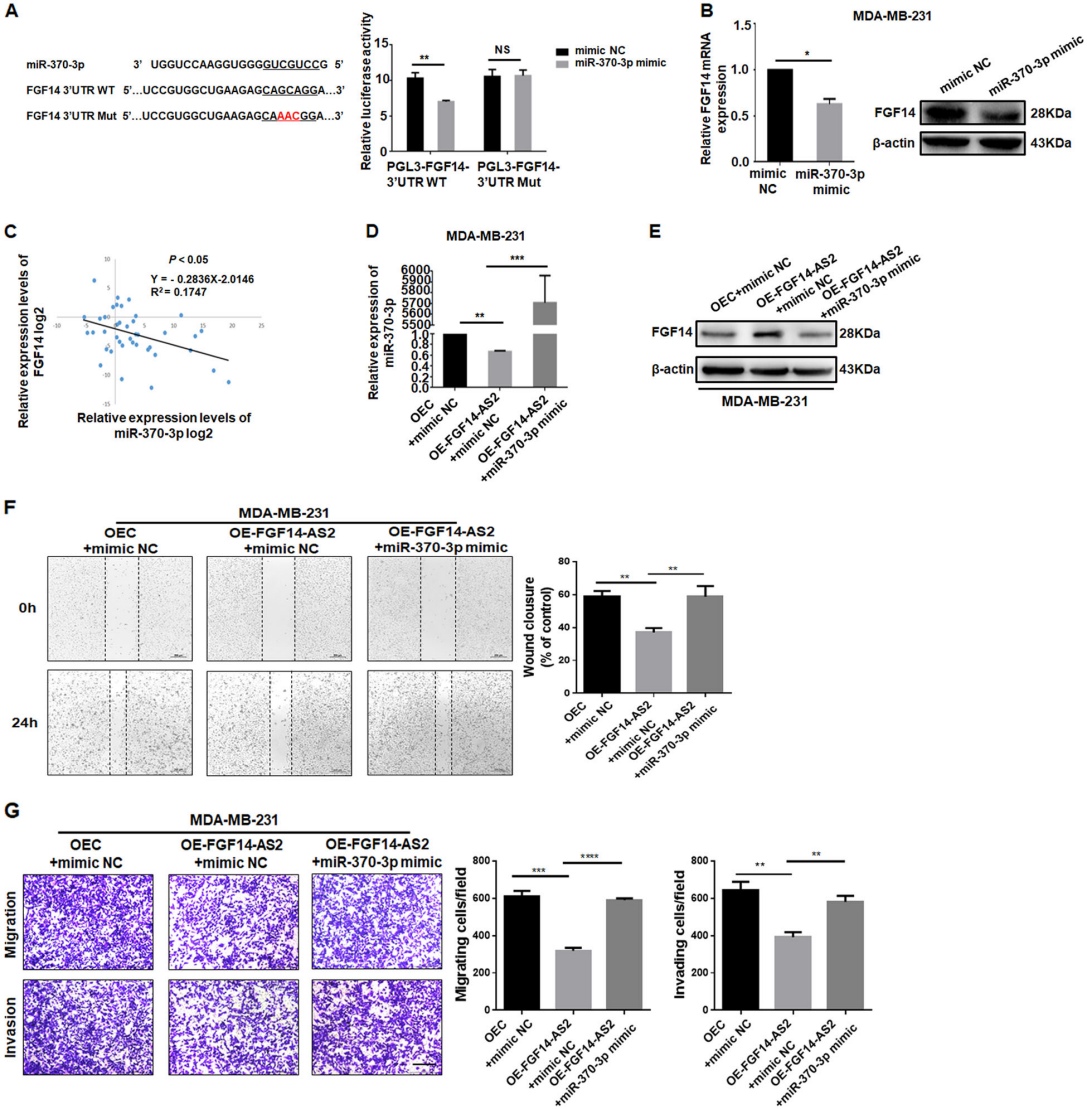
FGF14-AS2 regulates FGF14 expression by sponging miR-370-3p. **a** Left: The putative miR-370-3p binding site in the 3′-UTR of FGF14 and the designed mutant sequence. Right: Luciferase reporter assay in HEK 293T cells transfected with pGL3-FGF14-3′-UTR (WT or Mut) and miR-370-3p mimic. **b** Relative FGF14 mRNA and protein levels in MDA-MB-231 cells transfected with miR-370-3p mimic or its negative control. **c** Spearman correlation analysis of the association between miR-370-3p and FGF14 expression levels in 45 breast cancer tissues. **d**, **e** qRT-PCR determination of miR-370-3p (**d**) and western blotting analysis of FGF14 (**e**) expression levels in MDA-MB-231 cells co-transfected with pLVX-FGF14-AS2 and miR-370-3p mimic, or with pLVX-FGF14-AS2 and mimic NC. **f** Wound healing assays of the migration abilities of MDA-MB-231 cells after co-transfection with pLVX-FGF14-AS2 and miR-370-3p mimic, or with pLVX-FGF14-AS2 and mimic NC. Scale bar: 200 μm. **g** Transwell assays of the migratory and invasive abilities of MDA-MB-231 cells after co-transfection with pLVX-FGF14-AS2 and miR-370-3p mimic, or with pLVX-FGF14-AS2 and mimic NC. Scale bar: 100 μm. Data are the mean ± SD of three independent experiments. *P < 0.05; **P < 0.01; ***P < 0.001; ****P < 0.0001. NS, no significant difference.

According to the prediction data, there is a putative miR-370-3p binding site in the FGF14 3′-UTR (Fig. 6a, left), suggesting that FGF14 might be a target of miR-370-3p. Dual-luciferase reporter assay was conducted in HEK 293T cells to verify their binding relationship. Ectopic expression of miR-370-3p suppressed FGF14-3′-UTR-WT luciferase activity significantly, but did not affect that of FGF14-3′-UTR-Mut (Fig. 6a, right). As expected, forced expression of miR-370-3p reduced FGF14 expression significantly at both the mRNA and protein level in MDA-MB-231 cells (Fig. 6b). Furthermore, miR-370-3p expression correlated negatively with FGF14 level in breast cancer tissues (Fig. 6c). These data suggest that FGF14 is a direct target of miR-370-3p.

Considering the regulatory relationship between FGF14-AS2 and miR-370-3p, and miR-370-3p and FGF14, we hypothesized that FGF14-AS2 might modulate FGF14 expression by sponging miR-370-3p. Indeed, miR-370-3p mimic reversed the upregulation of FGF14 protein induced by FGF14-AS2 effectively (Fig. 6d, e). Moreover, wound healing and Transwell assays revealed that miR-370-3p mimic prominently reversed the decreased cell migration and invasion caused by FGF14-AS2 overexpression (Fig. 6f, g). Taken together, these data strongly support the hypothesis that highly expressed FGF14-AS2 inhibits cell migration and invasion in breast cancer by upregulating FGF14 via sponging miR-370-3p.

## Discussion

Recently, dozens of lncRNAs have been identified as critical players in cancer occurrence and metastasis. Here, we determined that the lncRNA FGF14-AS2 was downregulated in human breast cancer tissues by analyzing two independent GEO datasets (GSE29431 and GSE54002). We validated this finding in a cohort of 45 paired breast cancer and nontumorous tissue samples. We found that FGF14-AS2 expression level correlated negatively with TNM stage, but was not associated with age, ER status, PR status, or HER2 status. In vitro assays demonstrated that FGF14-AS2 knockdown promoted cell migration and invasion, whereas FGF14-AS2 overexpression impaired it. Consistent with these findings, FGF14-AS2 overexpression suppressed the lung metastasis of breast cancer in vivo.

Antisense lncRNA can regulate the protein expression of complementary genes^21,22,28^. Accordingly, we studied the relationship between FGF14-AS2 and its coding counterpart, FGF14. There was a positive correlation between FGF14-AS2 and FGF14 in breast cancer tissues. In addition, FGF14-AS2 regulated FGF14 expression positively in breast cancer cells. FGF14, as a member of the nonsecreted intracellular FGF family, does not function as an FGF ligand^29^. Previous studies on FGF14 mainly focused on its functions in neural cells^30,31^. Interestingly, a recent study revealed that FGF14 is repressed in patients with nasopharyngeal carcinoma, and FGF14 overexpression suppresses cell proliferation^32^. In this study, we found that FGF14 was significantly downregulated in breast cancer tissues compared with noncancerous tissues. Moreover, we reveal for the first time that FGF14 knockdown promoted breast cancer cell migration and invasion, whereas FGF14 overexpression repressed it. Rescue experiments indicated that knockdown of FGF14 could partially reverse the inhibitory effects induced by FGF14-AS2, suggesting that FGF14-AS2-suppressed breast cancer cell migration and invasion was at least partially FGF14-dependent. We previously found that endogenous FGF14 is mainly localized to the nucleus in MDA-MB-231 and MCF-7 cells (unpublished data), which is consistent with the findings of Peled et al.^33^ In addition, FGF14 is predicted to bind DNA, and the three residues in its N terminal are very important for its DNA-binding ability^33^. As a DNA-binding protein, FGF14 may regulate the transcription of metastasis-associated genes directly or modulate the expression of metastasis-associated genes indirectly, thereby suppressing migration and invasion of breast cancer cells.

A growing number of reports have suggested that the cellular location of lncRNAs is important for their biological functions. Generally, cytoplasmic lncRNAs influence cellular signaling cascades and modulate mRNA stability or translation, whereas nuclear lncRNAs are responsible for RNA processing, transcriptional regulation, and chromatin interactions^9,16,34^. Here, we identified that FGF14-AS2 was mainly located in the cytoplasm of breast cancer cells and could interact with AGO2, which suggests that FGF14-AS2 may function as an endogenous miRNA sponge. Bioinformatics analyses and luciferase reporter assays revealed that FGF14-AS2 interacted with miR-370-3p. Moreover, FGF14-AS2 downregulated miR-370-3p expression. However, the expression level of pri-miR-370-3p was not altered by FGF14-AS2, suggesting that FGF14-AS2 could not regulate the transcription of miR-370-3p. We speculate that FGF14-AS2 may regulate miR-370-3p expression via influencing its transport, processing, and/or stability.

The aberrant miR-370-3p expression has been reported in various cancers. In most cases, miR-370-3p is lowly expressed and plays a tumor-suppressive role in bladder cancer^24^, hepatocellular carcinoma^25^, laryngeal squamous cell carcinoma^35^, osteosarcoma^36^, glioma^37^, and ovarian cancer^38^. However, cumulative evidence indicates that miR-370-3p expression is upregulated in gastric carcinoma^26^ and melanoma^27^, suggesting that it might act as an oncogene. In this study, we found that miR-370-3p was significantly highly expressed in breast cancer tissues compared with their matched noncancerous tissues. In addition, inhibiting miR-370-3p suppressed breast cancer cell migration and invasion, whereas miR-370-3p overexpression had the opposite effects. Furthermore, miR-370-3p expression was negatively associated with FGF14-AS2 expression in breast cancer tissues. Moreover, miR-370-3p overexpression downregulated FGF14-AS2 expression, while miR-370-3p knockdown caused an opposite change in FGF14-AS2 expression, indicating mutual regulation between miR-370-3p and FGF14-AS2. Ballantyne et al.^39^ have demonstrated that some miRNAs bound to lncRNAs, with the aid of other RNA-binding proteins, to regulate lncRNA stability^39^. In addition, we think that the genes encoding enzymes or proteins responsible for the biogenesis of lncRNA may also be regulated by miRNAs.

Recent studies have revealed that some lncRNAs acted as ceRNAs of specific miRNAs, and then target other terminal mRNAs in the cytoplasm^40–42^. Using online prediction software, we show that FGF14 is a potential miR-370-3p target. Luciferase reporter assays verified that miR-370-3p targeted FGF14 mRNA at its 3′-UTR. As expected, miR-370-3p overexpression inhibited FGF14 mRNA and protein expression. Moreover, miR-370-3p expression levels correlated negatively with FGF14 in breast cancer tissues. In addition, miR-370-3p mimic could partially reverse the FGF14 protein upregulation induced by FGF14-AS2 and weaken the suppressive effects of FGF14-AS2 on breast cancer cell migration and invasion. These data strongly support the hypothesis that highly expressed FGF14-AS2 suppresses breast cancer cell migration and invasion by upregulating FGF14 via sponging miR-370-3p.

Although the above findings and a previous study have shown that FGF14-AS2 is downregulated in breast cancer^20^, the mechanism for FGF14-AS2 dysregulation in breast cancer remains unclear. A few studies have revealed that epigenetic modification can regulate lncRNA transcription. For example, EZH2 suppresses lncRNA SPRY4-IT1 expression in non-small cell lung cancer cells^43^; DNMT1-mediated DNA methylation silences MEG3 expression in gliomas^44^. Here, genome bioinformatics analysis of the FGF14-AS2 promoter sequence identified a CpG island in the promoter (Figure S9). We also analyzed the FGF14-AS2 promoter methylation status in breast cancer cell lines (MCF-7, MDA-MB-453, and MDA-MB-231) and the normal mammary epithelial cell line HBL-100 using methylation-specific PCR. The CpG island was hypermethylated in all three breast cancer cell lines and the normal mammary epithelial cells, and there were no obvious differences in FGF14-AS2 promoter methylation levels among these cells (data not shown), suggesting that FGF14-AS2 promoter hypermethylation might not be the major reason for its dysregulation in breast cancer. In addition to epigenetic modification, several key transcription factors can also contribute to lncRNA dysregulation in human cancer cells, such as E2F1^45^, p53^46^, and SP1^47,48^. However, the exact regulatory mechanisms of FGF14-AS2 downregulation in breast cancer still require further exploration.

**Fig. 7:**
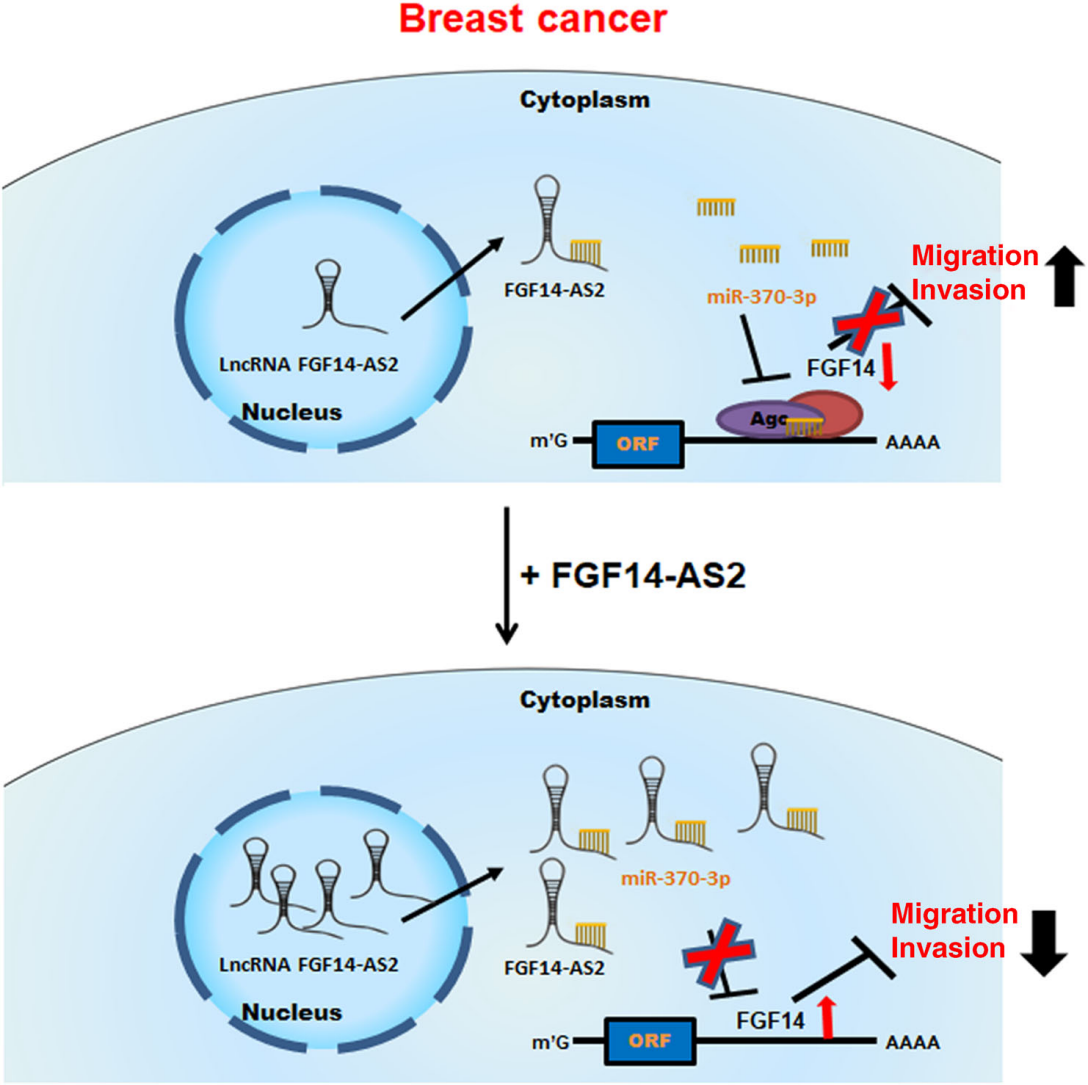
Schematic diagram illustrating the roles of FGF14-AS2 in breast cancer cells. FGF14-AS2 functions as a tumor suppressor by sponging miR-370-3p, weakening the suppressive effect of miR-370-3p on FGF14, thereby inhibiting cell migration and invasion.

In summary, we demonstrate that FGF14-AS2 functions as a tumor suppressor by sponging miR-370-3p, weakening the suppressive effect of miR-370-3p on FGF14, thereby inhibiting cell migration and invasion (Fig. 7). Our data indicate that the FGF14-AS2/miR-370-3p/FGF14 axis functions as an important player in breast cancer metastasis, which may be a novel target in breast cancer therapy.

## Materials and methods

### Tissue samples

Forty-five pairs of breast cancer and adjacent nontumor tissues were obtained from patients who had been diagnosed with breast cancer based on histopathologic evaluation and who had undergone surgery at the First Affiliated Hospital of Nanjing Medical University between 2014 and 2016. All patients did not receive chemotherapy or radiotherapy before surgery. All samples were immediately snap-frozen in liquid nitrogen and stored at −80 °C until used. The Research Ethics Committee of Nanjing Medical University approved the study, and written consent was obtained from each patient enrolled in the study.

### Cell culture and transfection

Human breast cancer cell lines (MCF-7, MDA-MB-453, MDA-MB-231, and HCC-1937) and a normal mammary epithelial cell line (HBL-100) were purchased from American Type Culture Collection (Manassas, VA, USA). All cells were cultured in Dulbecco’s modified Eagle’s medium (Gibco, Carlsbad, CA, USA) supplemented with 10% fetal bovine serum (FBS, Gibco) and 1% penicillin/streptomycin (Invitrogen, Carlsbad, CA, USA) in a humidified atmosphere with 5% CO_2_ at 37 °C. Cells have been authenticated by STR profiling and tested for mycoplasma contamination. MiR-370-3p mimic, miR-370-3p inhibitor, their respective controls, small interfering RNA (siRNA) against FGF14 expression (si-FGF14), and negative control siRNA (si-NC) were chemically synthesized by RiboBio (Guangzhou, China). The FGF14-overexpressing plasmid pCMV3-FGF14-Flag and empty vector were from Sino Biological Inc. (Beijing, China). Cells were plated in 6-well plates at 60–70% confluence one day prior to transfection. Cell transfection was conducted using Lipofectamine 2000 (Invitrogen) in accordance with the manufacturer’s instructions. Table S2 lists the siRNA sequences.

### Plasmid construction and lentivirus preparation

A construct overexpressing FGF14-AS2 was generated by ligating the full-length human FGF14-AS2 into vector pLVX-EF1α-IRES-puro (Clontech, Mountain View, CA, USA). For FGF14-AS2 knockdown, shRNA oligos against FGF14-AS2 were synthesized, annealed, and ligated into vector pLentilox 3.7 (Addgene, Cambridge, MA, USA), with a nontargeting control sequence (shNC) as the control. Table S3 lists the primers used. High-titer lentivirus was packaged in HEK 293T cells. The viral particles were collected by centrifugation at 48 h post transfection, and applied to cells in the presence of 5 μg/mL polybrene for 48 h. FGF14-AS2-overexpressing cells were selected using puromycin (5 μg/mL) for 2 weeks. Gene knockdown and overexpression were confirmed by qRT-PCR.

### Total RNA isolation and qRT-PCR

Total tissue and cellular RNA were isolated using TRIzol (Invitrogen), and complementary DNA was synthesized from 1 μg total RNA using the PrimeScript RT reagent (Takara, Otsu, Japan) following the manufacturer’s instructions. The expression levels of mature miRNAs were detected by stem-loop RT-PCR as described by Chen et al.^49^ Briefly, stem-loop RT primer bound to miRNA molecules and cDNA was synthesized with reverse transcriptase. Then, the RT products were amplified using a miRNA-specific forward primer and the universal reverse primer. The real-time PCR was performed using FastStart Universal SYBR Green Master (Roche, Indianapolis, IN, USA) in a Roche LightCycler 96 Real-Time PCR System. The amplification conditions were 95 °C for 10 min, followed by 40 amplification cycles of 95 °C for 10 s and 60 °C for 30 s. The mRNA expression values were normalized to that of the β-actin (*ACTB*) gene; *U6* was used as an endogenous control for miRNA expression. Tables S4 and S5 list the primer sequences.

### Western blotting

Cells were washed with phosphate-buffered saline (PBS) and harvested in radioimmunoprecipitation assay buffer (Beyotime, Haimen, China) supplemented with a protease inhibitor cocktail (Roche). Equal amounts of protein were loaded on a 10% sodium dodecyl sulfate (SDS)-polyacrylamide gel for electrophoresis, and transferred to polyvinylidene difluoride membranes (Millipore, Billerica, MA, USA). The membranes were probed with specific antibodies overnight at 4 °C, and then incubated with secondary antibodies at room temperature for 1 h. Each protein band was visualized by ECL chemiluminescent reagent (Millipore). The following primary antibodies were used: β-actin (cat. no. sc47778, Santa Cruz, Dallas, TX, USA), FGF14 (cat. no. A6588, ABclonal, Wuhan, China), and Flag (cat. no. F3165, Sigma-Aldrich, St. Louis, MO, USA).

### Cell proliferation assay

The viability of breast cancer cells was monitored using Cell Counting Kit-8 (Beyotime) according to the manufacturer’s protocol. Cells were seeded in 96-well plates at an initial density of 2 × 10^3^ cells per well. At day 0, 1, 2, and 3, the cells were treated with CCK-8 reagents at 37 °C for 2 h and the optical density values were detected at 450 nm with a microplate reader (BioTek, Winooski, VT, USA). All experiments were repeated three times in six replicates.

### Wound healing assay

Cells were seeded in 6-well plates and grown to 90% confluence, and then a linear wound was scratched in the cell monolayer with a pipette tip. Separated cells were washed out using PBS. Wounded cultures were incubated in serum-free medium, and the edges of the scratch were photographed. Random migration was evaluated by measuring the area of occupancy with Image-Pro Plus (Media Cybernetics, Rockville, MD, USA).

### Transwell migration and invasion assays

Transwell chambers (BD Biosciences, Bedford, MA, USA) were coated (for invasion assay) or uncoated (for migration assay) with Matrigel (BD Biosciences). A total of 5 × 10^4^ cells were plated onto the upper chamber for the migration assay, and 1 × 10^5^ cells were added to the upper chamber for the invasion assay. Total culture medium containing 10% FBS was added to the lower chamber. After incubation for 20 or 40 h, cells that had migrated and invaded through the membrane to the lower surface were fixed with methanol and stained with crystal violet. The stained cells were photographed and counted under light microscopy (magnification, ×100) in four randomly selected fields per membrane.

### In vivo tumor metastasis assays

Female athymic BALB/c nu/nu mice (4 weeks old) were manipulated and cared for according to the National Institutes of Health Guide for the Care and Use of Laboratory Animals. The Committee on the Ethics of Animal Experiments of the Nanjing Medical University, Nanjing, China, approved the protocol. The nude mice were maintained under pathogen-free conditions and randomly divided into two groups (5 mice per group). Cell aliquots (100 μL) were injected into the lateral tail veins of the mice. After 6 weeks, lungs were excised and photographed, and visible nodules on the lung surface were counted and collected for subsequent analysis.

### H&E staining and immunohistochemistry

The dissected lungs were fixed overnight in 4% paraformaldehyde, embedded in paraffin, and then sectioned at 5-μm width. Lungs were stained with H&E according to the standard protocols. For immunohistochemical staining, lungs were deparaffinized, rehydrated through graded alcohol, and washed with PBS. Samples were blocked with 2% bovine serum albumin at room temperature for 1 h. The sections were treated with FGF14 rabbit polyclonal antibody at 4 °C overnight, and then incubated with secondary antibody at 37 °C for 30 min. The sections were then stained with diaminobenzidine (Sigma-Aldrich) and counterstained with hematoxylin (Sigma-Aldrich).

### Fluorescence in situ hybridization

MDA-MB-231 cells were fixed in 4% formaldehyde for 10 min and washed with PBS. Then, the cells were permeabilized in 0.5% Triton X-100 at 4 °C for 5 min, washed with PBS, and pre-hybridized at 37 °C for 30 min before hybridization. The anti-FGF14-AS2 (cat. no. lnc1100313), anti-U6 (cat. no. lnc110101), and anti-18s (cat. no. lnc110102) oligodeoxynucleotide probes were used in the hybridization solution at 37 °C overnight in the dark. The next day, the cells were counterstained with 4′, 6-diamidino-2-phenylindole and imaged using a confocal laser scanning microscope. The RNA FISH probes were designed and synthesized by RiboBio.

### RNA immunoprecipitation

RIP was performed using an EZ-Magna RIP Kit (Millipore) according to the manufacturer’s protocol. Briefly, whole-cell lysate was incubated with RIP buffer containing magnetic beads conjugated with anti-AGO2 antibody or negative control IgG for 6 h at 4 °C. The beads were washed and then incubated with 0.1% SDS/0.5 mg/mL proteinase K for 30 min at 55 °C to remove proteins. Finally, the immunoprecipitated RNA underwent qRT-PCR to detect the enrichment of FGF14-AS2 and miR-370-3p.

### Dual-luciferase reporter assay

The potential miR-370-3p binding sites in FGF14-AS2 were predicted using RNAhybrid (http://bibiserv2.cebitec.uni-bielefeld.de/rnahybrid). The WT FGF14-AS2 sequence and its mutant (Mut, only the putative miR-370-3p binding sites were mutated) were synthesized and cloned into vector psiCHECK-2 (Promega, Madison, WI, USA), respectively. MiR-370-3p and miR-761 target genes were predicted using TargetScan (http://www.targetscan.org/) and miRDB (http://www.mirdb.org/). The FGF14 3′-UTR containing the predicted potential miR-370-3p and miR-761 binding sites was amplified and inserted into vector pGL3-Promoter (Promega). pGL3-FGF14-3′-UTR-Mut was generated by site-directed mutagenesis, replacing the three ribonucleotides of the miR-370-3p complementary sequence. Luciferase and Renilla activities were assessed using a Dual-Luciferase Reporter Assay Kit (Promega). The detected luciferase activity was normalized to the Renilla luciferase activity. Table S3 lists the primers used.

### Statistical analysis

The adequate sample size was determined according to the previous studies that performed analogous experiments. For animal studies, no blinding was used. All assays were implemented thrice. Statistical analysis was performed using SPSS 19.0 (SPSS, Chicago, IL, USA). Results were expressed as the mean ± SD. Comparisons between two groups were analyzed using a two-sided Student’s *t* test. The variances between the groups that are being statistically compared were similar. Correlation between FGF14-AS2 expression and clinicopathological characteristics of the patients with breast cancer were examined using the *χ*^2^ test. The relationship between FGF14-AS2, miR-370-3p, and FGF14 expression levels were assessed using Spearman correlation analysis. *P* < 0.05 was considered statistically significant.

## Supplementary information

Figure S1

Figure S2

Figure S3

Figure S4

Figure S5

Figure S6

Figure S7

Figure S8

Figure S9

Supplemental figure legends

Supplemental tables

## Data Availability

The datasets used or analyzed during the current study are available from the corresponding author on reasonable request. All data generated or analyzed during this study are included in this published article and its Supplementary information files.
