# Supplementary figures and images for: Long noncoding RNA FGF14-AS2 inhibits breast cancer metastasis by regulating the miR-370-3p/FGF14 axis
Source: Cell Death Discov. 2020 Oct 12;6:103. doi: 10.1038/s41420-020-00334-7 (PMC7548970; doi:10.1038/s41420-020-00334-7)

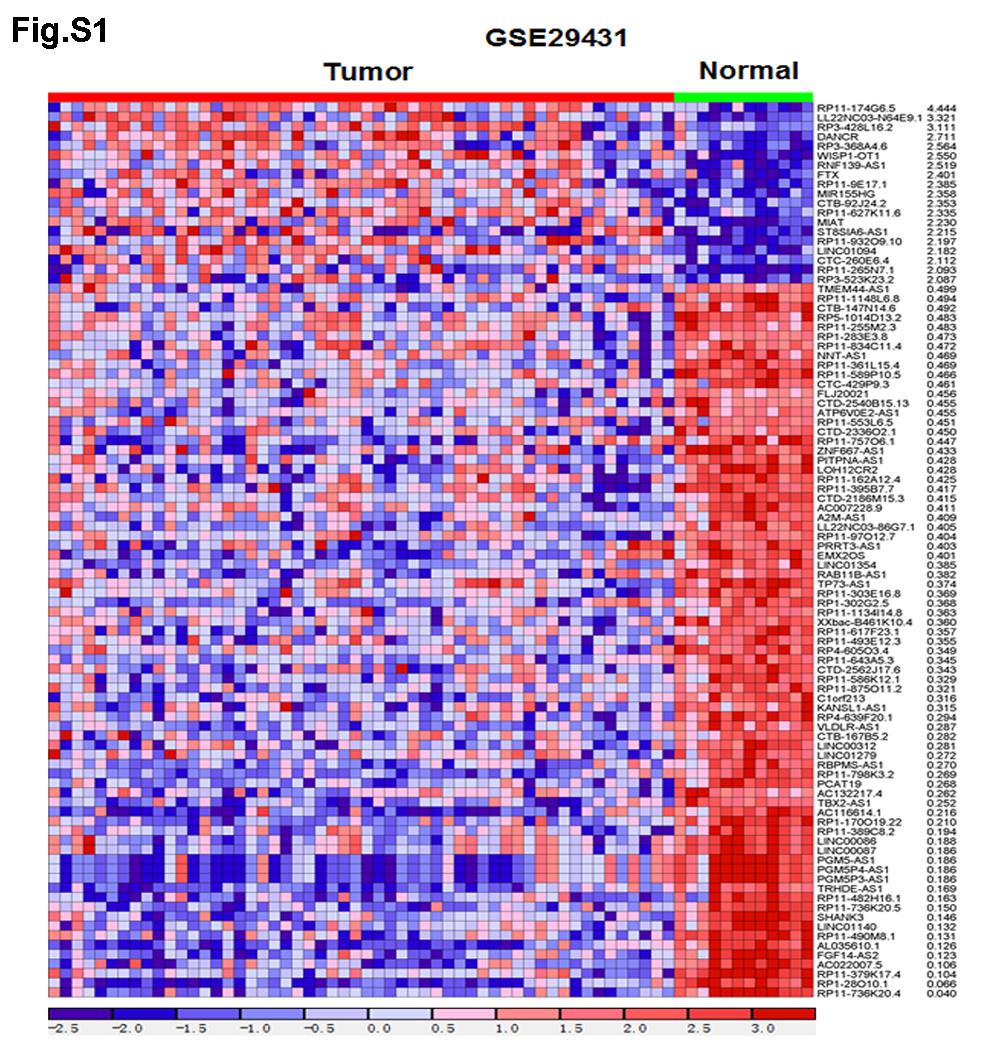

Supplement: Supplementary file 1 — Figure S1 [file 41420_2020_334_MOESM1_ESM.tif]

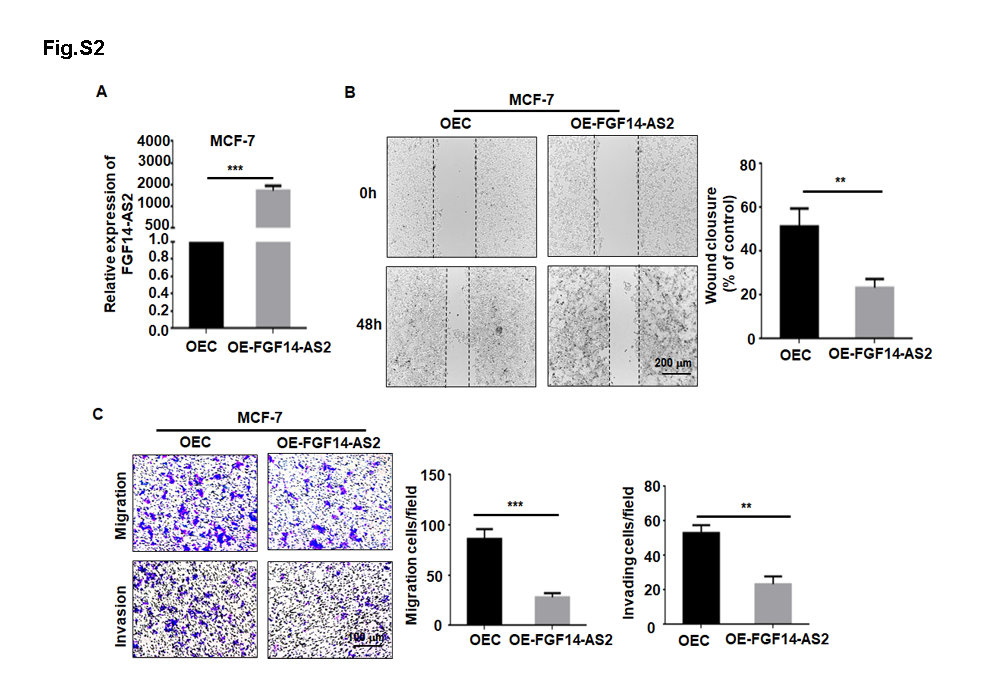

Supplement: Supplementary file 2 — Figure S2 [file 41420_2020_334_MOESM2_ESM.tif]

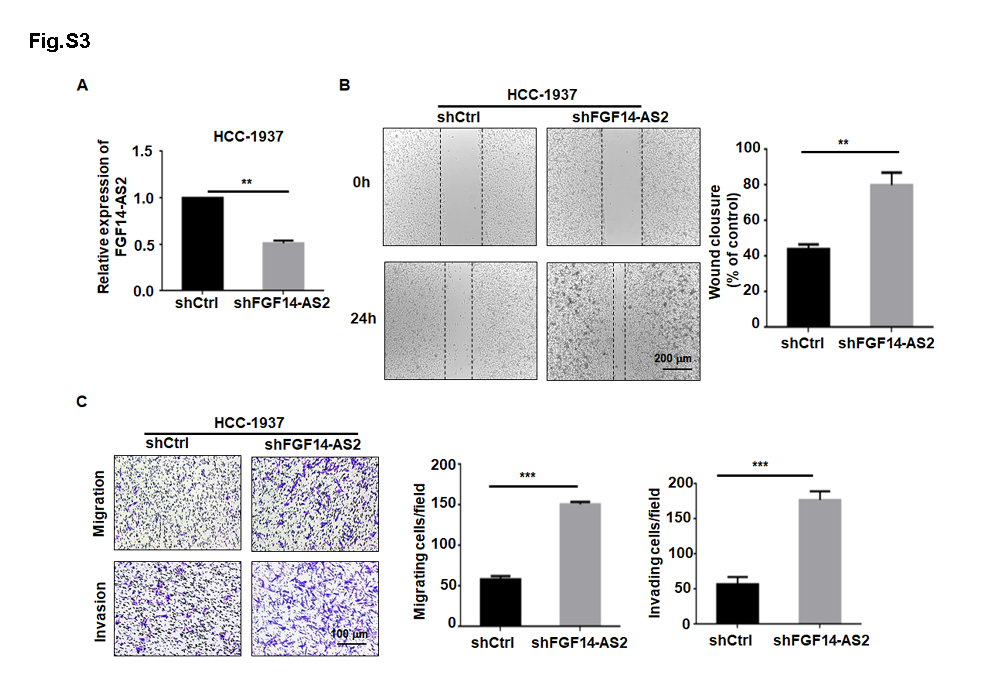

Supplement: Supplementary file 3 — Figure S3 [file 41420_2020_334_MOESM3_ESM.tif]

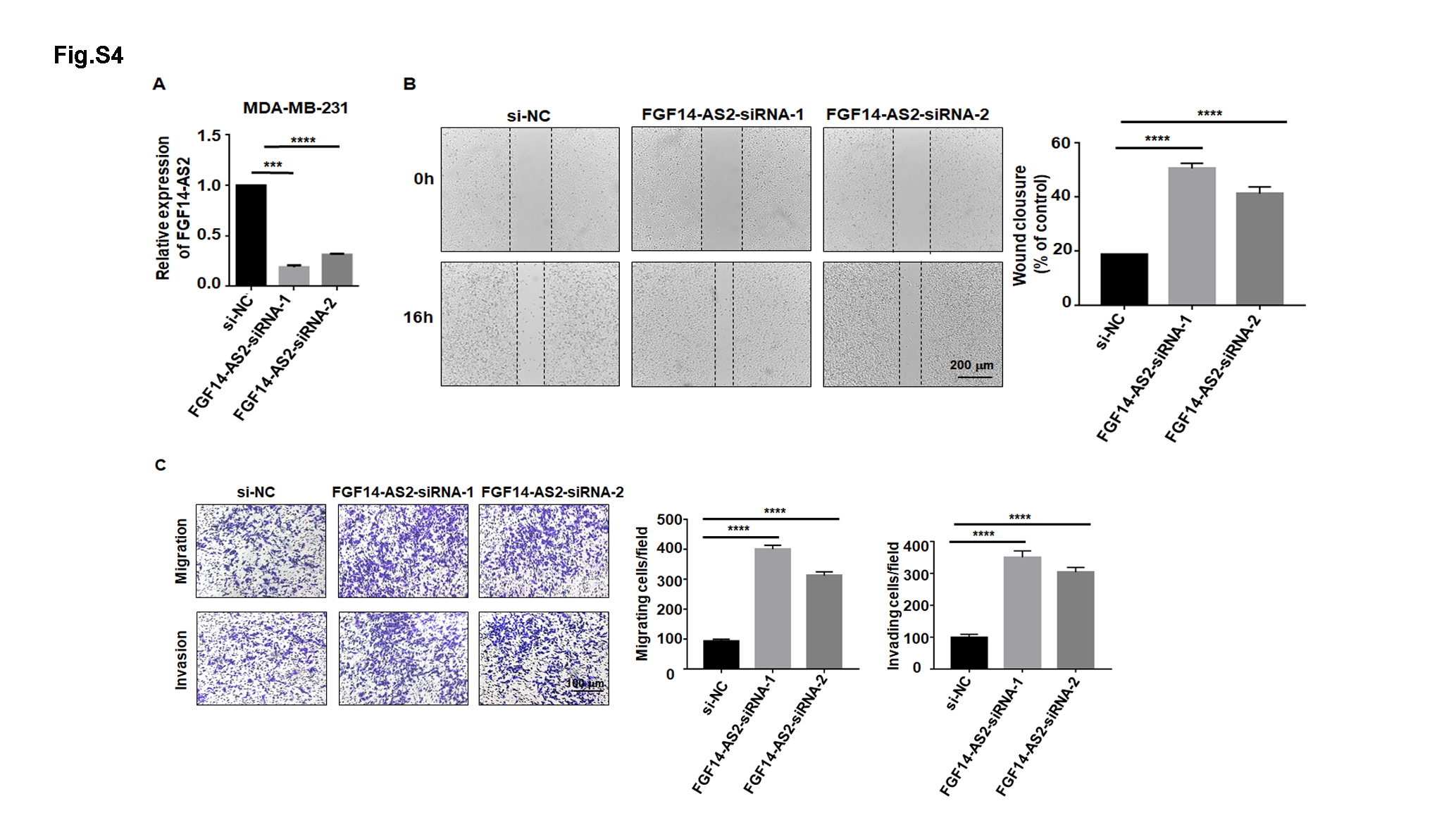

Supplement: Supplementary file 4 — Figure S4 [file 41420_2020_334_MOESM4_ESM.tif]

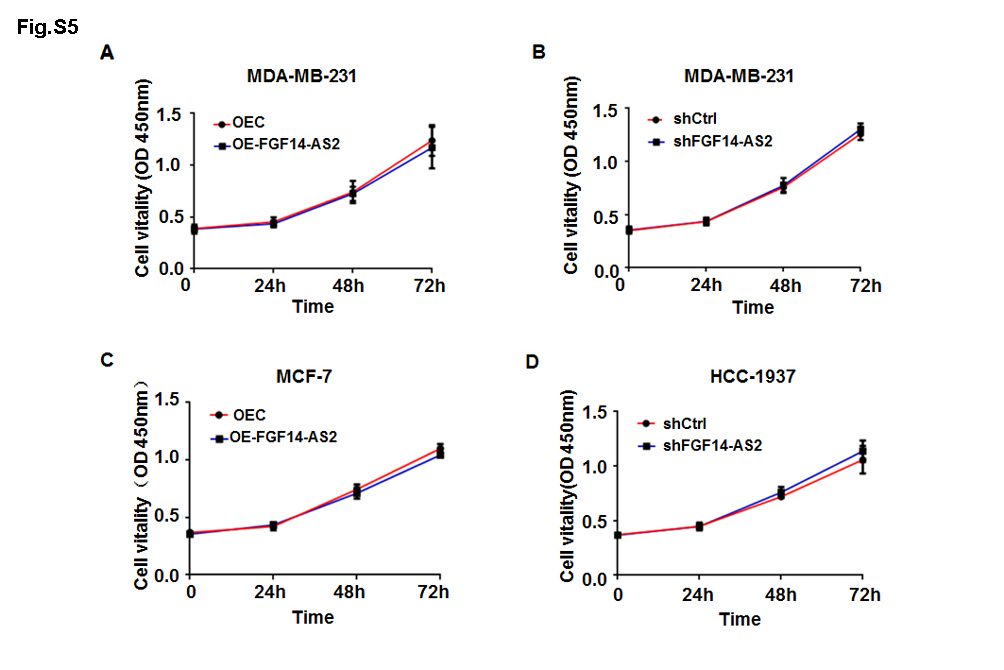

Supplement: Supplementary file 5 — Figure S5 [file 41420_2020_334_MOESM5_ESM.tif]

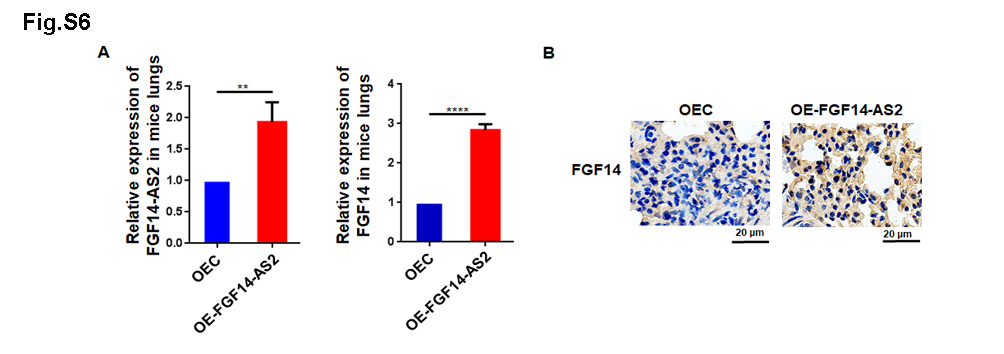

Supplement: Supplementary file 6 — Figure S6 [file 41420_2020_334_MOESM6_ESM.tif]

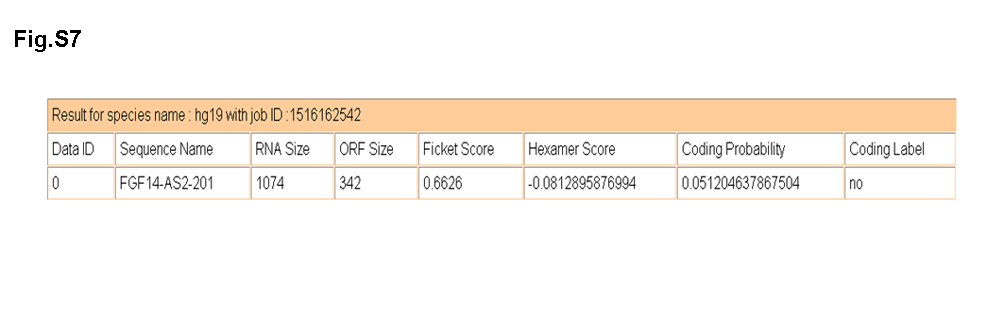

Supplement: Supplementary file 7 — Figure S7 [file 41420_2020_334_MOESM7_ESM.tif]

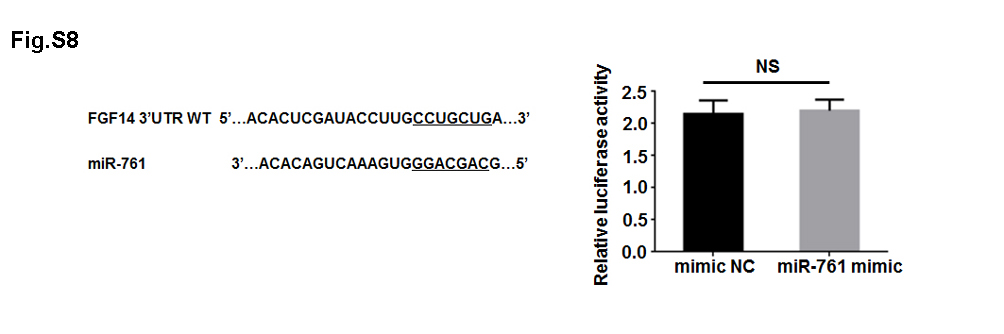

Supplement: Supplementary file 8 — Figure S8 [file 41420_2020_334_MOESM8_ESM.tif]

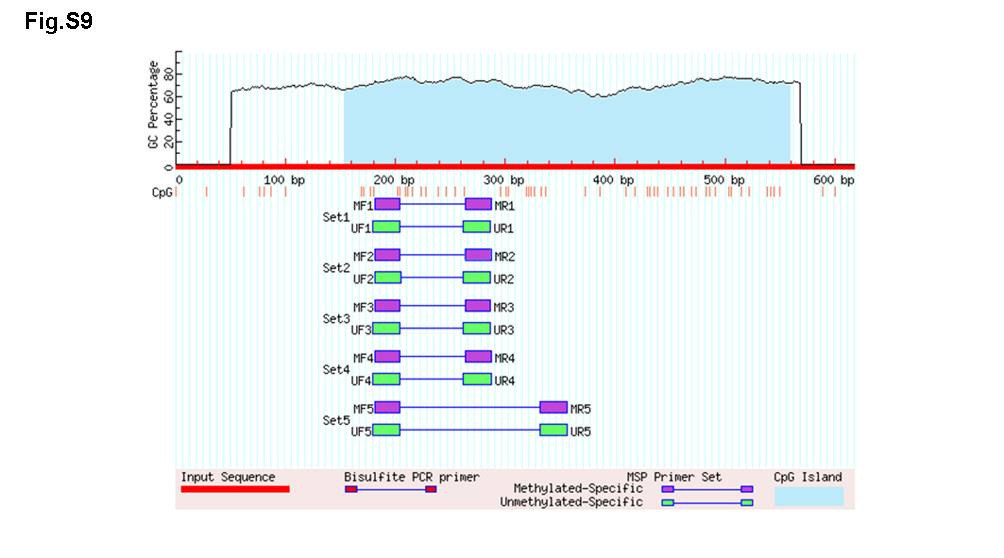

Supplement: Supplementary file 9 — Figure S9 [file 41420_2020_334_MOESM9_ESM.tif]
