# Supplementary material for: Long noncoding RNA FGF14-AS2 inhibits breast cancer metastasis by regulating the miR-370-3p/FGF14 axis
Source: Cell Death Discov. 2020 Oct 12;6:103. doi: 10.1038/s41420-020-00334-7 (PMC7548970; doi:10.1038/s41420-020-00334-7)
Supplement: Supplementary file 10 — Supplemental figure legends [file 41420_2020_334_MOESM10_ESM.docx]

**Supplemental figure legends**

**Figure S1** The hierarchical clustering heat map of the top 94 lncRNAs differentially expressed between breast cancer (Tumor) and adjacent non-tumorous samples (Normal) from the GSE29431 dataset (fold change > 2; *P* < 0.05).

**Figure S2** Overexpression of FGF14-AS2 suppresses MCF-7 cell migration and invasion. (A) qRT-PCR analysis of the efficiency of FGF14-AS2 expression in stable FGF14-AS2 overexpression MCF-7 cells. (B) Wound healing assay was performed to determine the migration ability of FGF14-AS2 overexpression MCF-7 cells. (C) Transwell assay was performed to determine the migration and invasive abilities of FGF14-AS2 overexpression MCF-7 cells. 5 × 10^4^ cells were plated onto the upper chamber for migration assays, and 1 × 10^5^ cells were added to the upper chamber for invasion assays. Total culture medium containing 10% FBS was added to the lower chamber. After incubation for 48 h, cells that had migrated and invaded through the membrane to the lower surface were fixed with methanol and stained with crystal violet. ^**^*P* < 0.01; ^***^*P* < 0.001. OEC: overexpression control cells; OE-FGF14-AS2: FGF14-AS2 overexpression cells.

**Figure S3** Silencing FGF14-AS2 promotes HCC-1937 cell migration and invasion. (A) qRT-PCR analysis of the efficiency of FGF14-AS2 expression in stable FGF14-AS2 knockdown HCC-1937 cells. (B) Wound healing assay was performed to determine the migration ability of FGF14-AS2 knockdown HCC-1937 cells. (C) Transwell assay was performed to determine the migration and invasive abilities of FGF14-AS2 knockdown HCC-1937 cells. 1 × 10^4^ cells were plated onto the upper chamber for migration assays, and 5 × 10^4^ cells were added to the upper chamber for invasion assays. Total culture medium containing 10% FBS was added to the lower chamber. After incubation for 16 h or 24 h, cells that had migrated and invaded through the membrane to the lower surface were fixed with methanol and stained with crystal violet. ^**^*P* < 0.01; ^***^*P* < 0.001. shCtrl: knockdown control cells; shFGF14-AS2: FGF14-AS2 knockdown cells.

**Figure S4** Silencing FGF14-AS2 promotes MDA-MB-231 cell migration and invasion. (A) qRT-PCR analysis of the efficiency of FGF14-AS2 expression in FGF14-AS2 siRNA transfected MDA-MB-231 cells. (B) Wound healing assay was performed to determine the migration ability of FGF14-AS2 siRNA transfected MDA-MB-231 cells. (C) Transwell assay was performed to determine the migration and invasive abilities of FGF14-AS2 siRNA transfected MDA-MB-231 cells. 2 × 10^4^ cells were plated onto the upper chamber for migration assays, and 1 × 10^5^ cells were added to the upper chamber for invasion assays. Total culture medium containing 10% FBS was added to the lower chamber. After incubation for 16 h or 24 h, cells that had migrated and invaded through the membrane to the lower surface were fixed with methanol and stained with crystal violet. ^***^*P* < 0.001; ^****^*P* < 0.0001.

**Figure S5** CCK-8 assay was used to determine the effect of FGF14-AS2 on the viability of MDA-MB-231 (A, B), MCF-7 (C), and HCC-1937 cells (D). OD: optical density; OEC: overexpression control cells; OE-FGF14-AS2: FGF14-AS2 overexpression cells; shCtrl: knockdown control cells; shFGF14-AS2: FGF14-AS2 knockdown cells.

**Figure S6** (A) qRT-PCR analysis of FGF14-AS2 and FGF14 levels in mice lungs (*n* = 5 per group). (B) Representative images of FGF14 immunohistochemical staining. Scale bar: 20 μm. Data are the mean ± SD. ^**^*P* < 0.01; ^****^*P* < 0.0001.

**Figure S7** The coding capability of FGF14-AS2 was determined by CPAT.

**Figure S8** Left: The putative miR-761 binding site in the FGF14 3′UTR (pGL3-FGF14-3’UTR). Right: Luciferase reporter assay of HEK 293T cells transfected with luciferase reporter plasmids pGL3-FGF14-3’UTR and miR-761 mimic. NS, no significant difference.

**Figure S9** Schematic representation of the predicted CpG island in FGF14-AS2 promoter region.
