# Supplementary material for: Long noncoding RNA FGF14-AS2 inhibits breast cancer metastasis by regulating the miR-370-3p/FGF14 axis
Source: Cell Death Discov. 2020 Oct 12;6:103. doi: 10.1038/s41420-020-00334-7 (PMC7548970; doi:10.1038/s41420-020-00334-7)
Supplement: Supplementary file 11 — Supplemental tables [file 41420_2020_334_MOESM11_ESM.docx]

**Supplemental tables**

**Table S1 Correlation between FGF14-AS2 expression and clinicopathological characteristics of breast cancer patients (n = 45)**

| **Clinicopathologic characteristics** | **FGF14-AS2** | | **P-value** |
| --- | --- | --- | --- |
|  | **Low expression** | **High expression** |  |
| **Age** |  |  | 0.448 |
| ≤ 60 | 26 | 7 |  |
| > 60 | 8 | 4 |  |
| **Clinical stage** |  |  | 0.021^*^ |
| I-II | 14 | 9 |  |
| III-IV | 20 | 2 |  |
| **ER** |  |  | 0.393 |
| Negative | 9 | 4 |  |
| Positive | 25 | 7 |  |
| **PR** |  |  | 0.330 |
| Negative | 11 | 5 |  |
| Positive | 23 | 6 |  |
| **HER2** |  |  | 0.535 |
| Negative | 8 | 2 |  |
| Positive | 26 | 9 |  |

^*^The values had statistical significant differences.

**Table S2 Sequences used for knockdown of target genes**

| **Names** | **sense/anti-sense** | **Sequences 5'-3'** |
| --- | --- | --- |
| si-NC | sense | UUCUCCGAACGUGUCACGUTT |
|  | anti-sense | ACGUGACACGUUCGGAGAATT |
| si-FGF14 | sense | GCCAUGUACCGAGAACCAUTT |
|  | anti-sense | AUGGUUCUCGGUACAUGGCTT |
| si-FGF14-AS2-1 | sense | GGAAUGUCUUCCUUGUUAATT |
|  | anti-sense | UUAACAAGGAAGACAUUCCTT |
| si-FGF14-AS2-2 | sense | UGGCAACUAUGAACCUAAUTT |
|  | anti-sense | AUUAGGUUCAUAGUUGCCATT |

**Table S3 Primers used for plasmid constructs**

| **Primer names** | **Forward/**  **Reverse** | **Sequences 5'-3'** |
| --- | --- | --- |
| pLVX-EF1α-IRES-puro-FGF14-AS2 | Forward | CGGAATTCGCGTAGCGAAGCCCCCG |
|  | Reverse | GCTCTAGAAGGTTTTCAATACACTTTA |
| pLentilox 3.7-shFGF14-AS2 | Forward | TTGGAGAAAAGCCTTGTTAACGCCAAATAGATCAGGGTGGTATTCAAGAGATACCACCC |
|  | Reverse | ATCGATACCGTCGACCTCGAGAAAAAGCCAAATAGATCAGGGTGGTATCTCTTGAATAC |
| pGL3-FGF14-3’UTR-WT | Forward | GCTCTAGACCAGATCCTCACAGGTGTTG |
|  | Reverse | GCTCTAGAGATGCAATTTGTAATAATAGGTTTAG |
| pGL3-FGF14-3’UTR-Mut | Forward | CGTGGCTGAAGAGCAAACGGAAGTAAGC |
|  | Reverse | GTTTGCTCTTCAGCCACGGAGCAGGAAT |

**Table S4 Primers used for qRT-PCR assay**

| **Gene names** | **Forward/Reverse** | **Sequences 5'-3'** |
| --- | --- | --- |
|  |  |  |
| hsa-FGF14-AS2 | Forward | ATTACCGAGGGGTTCCACGC |
|  | Reverse | GGTGTCCGGGACCAGAAAGT |
| hsa-FGF14 | Forward | CAAATGCACCCCGATGGAGC |
|  | Reverse | CCCTGGATGGCAACAACACG |
| β-actin | Forward | CCATGTACGTTGCTATCCAG |
|  | Reverse | CTTCATGAGGTAGTCAGTCAG |
| pri-miR-370-3p | Forward | GGGATGGGCGATAGTTCAGGTC |
|  | Reverse | TCACCCAAATCTTGGCTCCCTC |
| pri-miR-761 | Forward | CTTAGGTCTGCACCTGTTCAGC |
|  | Reverse | CGCTGCAGACTGGAGTAATTAG |

**Table S5 Primers used for stem-loop qRT-PCR assay**

| **Gene names** | **RT/Forward/**  **Reverse** | **Sequences 5'-3'** |
| --- | --- | --- |
|  |  |  |
| hsa-miR-370-3p | RT | GTCGTATCCAGTGCAGGGTCCGAGGTATTCGCACTGGATACGACACCAGG |
|  | Forward | GCCTGCTGGGGTGGAACC |
| hsa-miR-21-3p | RT | GTCGTATCCAGTGCAGGGTCCGAGGTATTCGCACTGGATACGACACAGCC |
|  | Forward | CAACACCAGTCGATGGG |
| hsa-miR-221-3p | RT | GTCGTATCCAGTGCAGGGTCCGAGGTATTCGCACTGGATACGACGAAACC |
|  | Forward | AGCTACATTGTCTGCTG |
| hsa-miR-224 | RT | GTCGTATCCAGTGCAGGGTCCGAGGTATTCGCACTGGATACGACCTAAAC |
|  | Forward | TCAAGTCACTAGTGGTTC |
| hsa-miR-3646 | RT | GTCGTATCCAGTGCAGGGTCCGAGGTATTCGCACTGGATACGACTGGGCT |
|  | Forward | AAAATGAAATGAGCCCAGCCCA |
| hsa-miR-761 | RT | GTCGTATCCAGTGCAGGGTCCGAGGTATTCGCACTGGATACGACTGTGTC |
|  | Forward | GCAGCAGGGTGAAACTGACACA |
| hsa-U6 | RT | AACGCTTCACGAATTTGCGT |
|  | Forward | CTCGCTTCGGCAGCACA |
|  | Reverse | AACGCTTCACGAATTTGCGT |
| hsa-miRNA | Reverse | CAGTGCAGGGTCCGAGGT |
